# Cryptocurrencies on the road to sustainability: Ethereum paving the way for Bitcoin

**DOI:** 10.1016/j.patter.2022.100633

**Published:** 2022-12-06

**Authors:** Alex De Vries

**Affiliations:** 1 School of Business and Economics, Vrije Universiteit Amsterdam, Amsterdam, the Netherlands; 2 Founder of Digiconomist, Almere, the Netherlands; 3 De Nederlandsche Bank, Amsterdam, the Netherlands

## Abstract

Amid the current climate emergency and global energy crisis, regulators have started to consider their options to limit the power demand of cryptocurrency networks. One specific way crypto-asset communities can limit their environmental impact is by avoiding or replacing the energy-intensive proof-of-work (PoW) mining mechanism. Ethereum, the second largest crypto-asset by market capitalization, had its PoW replaced with an alternative known as proof-of-stake during an event called The Merge on September 15, 2022. In this perspective, the likely range of electricity saved due to this change is estimated, while the limitations in assessing these figures are highlighted. Lastly, the challenges and opportunities in replicating The Merge on other cryptocurrencies such as Bitcoin are discussed.

## The bigger picture

Cryptocurrencies such as Bitcoin are facing increasing pressure from regulators around the world to limit their power demand. This perspective highlights how Ethereum, the second largest cryptoasset by market capitalization, likely succeeded in significantly reducing its power demand through an event called The Merge. This event occurred on September 15, 2022, and consisted of Ethereum’s proof-of-work mining mechanism being replaced with an alternative known as proof of stake. The Ethereum network likely reduced its power demand by 99.84% to 99.9996% as a result of this change. In absolute terms, the reduction in power demand could be equivalent to the electrical power requirement of a country such as Ireland or even Austria.

Even though it would still be premature for the Ethereum community to declare a complete victory over the sustainability concerns facing cryptoassets, the factors that contributed to the success of The Merge may now serve as a roadmap to enable a change from proof of work to proof of stake in Bitcoin and other cryptoassets still making use of proof of work.

## Teaser

Ethereum, the second largest cryptoasset by market capitalization, had its proof-of-work mining mechanism replaced with an alternative known as proof of stake during an event called The Merge on September 15, 2022. The Ethereum network likely reduced its power demand by 99.84% to 99.9996% as a result of this change. The merge may now serve as a roadmap to enable a change from proof of work to proof of stake in Bitcoin and other cryptoassets still making use of proof of work.

## Introduction

Over the past year, regulators worldwide have increased focus on the energy use and climate impacts of cryptocurrencies such as Bitcoin and Ethereum. Although estimates of these impacts vary, it has been suggested that the electric load demand of just the Bitcoin network could exceed 13 GW, with an associated carbon footprint of more than 65 megatons of CO_2_ (MtCO_2_) annually, as of 2021.^1^ This electric load demand would exceed half of the estimated power demand of all global data centers combined and represent nearly half a percent of the global electrical energy consumption. The estimated carbon footprint is also significant enough to exceed the estimated global reductions of CO_2_ by electric vehicles (51.9 MtCO_2_ in 2020^2^). Moreover, other cryptocurrencies may jointly add another 50% on top of Bitcoin’s energy hunger.^3^

Amid the current climate emergency and global energy crisis, regulators have started to consider their options to limit the power demand of these cryptocurrency networks. In some cases, this focus has already resulted in drastic actions. For example, during the spring of 2021, cryptocurrency mining bans were issued throughout China (previously housing a majority of the Bitcoin mining network), with environmental concerns being cited as the reason for doing so.^1^ Moreover, in March 2022, the European Parliament considered a potential ban on offering any kind of services related to cryptocurrencies making use of the energy-intensive mining process. The proposal was rejected in favor of additional environmental disclosure by cryptoasset service providers,^4^ but the European Central Bank later stated it was “highly unlikely” that European authorities would not pursue any further action (including the possibility of an outright ban) against cryptocurrency mining.^5^ In the United States, the state of New York is finishing new legislation to ban cryptocurrency miners from receiving behind-the-meter power from fossil fuel power plants.^6^ A report by the White House Office of Science and Technology Policy released in September 2022 recommended promoting “environmentally responsible crypto-asset technologies,” adding that legislation to “limit or eliminate” energy-intensive cryptocurrency mining should be considered if other measures to curb environmental impacts prove to be ineffective.^7^

One specific way cryptoasset communities can limit their environmental impact is by avoiding or replacing the energy-intensive mining process altogether. This perspective highlights how Ethereum, the second largest cryptoasset by market capitalization, likely succeeded in significantly reducing its power demand through an event called The Merge. This event occurred on September 15, 2022, and consisted of Ethereum’s proof-of-work (PoW) mining mechanism being replaced with an alternative known as proof of stake (PoS). In this perspective, the likely range of electricity saved due to this change is estimated, while the limitations in assessing these figures are highlighted. Lastly, the challenges and opportunities in replicating The Merge on other cryptocurrencies such as Bitcoin are discussed.

## PoW versus PoS

To understand how it is possible to significantly reduce the power demand of a cryptoasset by changing only a limited part of its software, one first needs to consider the role PoW and PoS have within the blockchain technology underpinning the Bitcoin and Ethereum networks and the different incentive structures they introduce. Regardless of whether PoW or PoS is being used, blockchains are literal chains of blocks of data. Operations (i.e., transactions) to update the current state of the network are processed within these blocks, which are then added to the end of the chain. In both Bitcoin and Ethereum, no single party is in charge of this process. Instead, they have open networks where, theoretically, anyone can join their computer hardware to assist in the block-creation process. In either case, a reward is provided for every created block as an incentive to participate. The difference between PoW and PoS becomes relevant with regard to the way in which blockchain-based networks align themselves on the current state of the network (i.e., the process of adding new blocks to the blockchain).

When a network employs PoW, the block-creation process is purposely made computationally difficult. New blocks can be added to the blockchain only once a valid PoW has been obtained, which can be achieved only through an iterative process of trial and error that can best be described as a numeric guessing game. A correct “guess” completes a block, allowing the lucky winner to add it to the blockchain and obtain the associated reward for doing so. The more guesses one can generate, the greater the chance of winning. The process repeats indefinitely after every newly created block. Before The Merge, the Ethereum network generated around 900 billion of these guesses every second of the day non-stop.^8^

In contrast, a network using PoS does not incentivize participants to compete on computational power to create new blocks for the blockchain. Instead, the selection process of which computers get to create the next block for the blockchain is primarily based on wealth. Participants have to acquire some of the native currency used on the respective blockchain network, which can then be locked up as collateral in the staking process. In Ethereum, a minimum of 32 units of the native cryptocurrency, Ether, are required to participate in this staking process. The software then randomly selects a “staker” to produce the next block for the blockchain. The greater the staked balance, the greater the chance of getting selected. While participants still need a device with sufficient storage capacity and an active internet connection, it is not relevant to the staking process how computationally powerful the device is.

## Estimated electricity savings

Although the lack of any incentive to compete on energy-intensive hardware is key to reducing a network’s power demand in switching from PoW to PoS, the exact effect this change had on Ethereum’s power requirement is not easy to determine. A major limitation in estimating the power demand of a PoW network is that even though it is possible to estimate the total computational power (known as the hashrate) in the network, the exact distribution of participating devices and their (overhead) energy costs is not known. The only figure that can be estimated with a high degree of certainty is the minimum power demand of the PoW network. This estimate can even be made on the back of an envelope, as it is calculated by multiplying the estimated computational power of the network with the power demand per unit of the computational power of the most power-efficient mining device available in the market. At the time of the Ethereum merge, on September 15, 2022, this device was Jasminer’s X4 with a maximum hashrate of 2.5 gigahashes per second at a power draw of 1,200 W.^9^ At an estimated total network hashrate of 871 terahashes per second on the day before The Merge,^8^ assuming this hashrate is solely coming from Jasminer X4 devices, the total power demand of the network would be at least 418 MW.

A significant downside of the Jasminer X4 is that it uses application-specific integrated circuits (ASICs), rendering the device useless for performing any task other than mining on the Ethereum mining algorithm. ASICs typically offer a significant competitive advantage in the mining process, but the inability to repurpose the device after The Merge may have repelled buyers. The device also did not have its release until November of 2021, leaving relatively little time before The Merge. It is therefore not unlikely that this device did not manage to gain much traction in the Ethereum mining industry compared with more generic computer components, such as a graphics processing units (GPUs), which, depending on the device type, could still be used to profitably mine Ethereum until The Merge (and be repurposed afterward). It has been generally assumed that a large (though unknown) part of the Ethereum network was comprised of GPUs. Reperforming the previous calculation with a top-performing GPU, such as the Nvidia RTX 3090Ti, with an estimated hashrate of 132 megahashes per second at 346 W,^10^ would already yield a total power demand of 2.23 GW instead of 418 MW.

**Figure 1:**
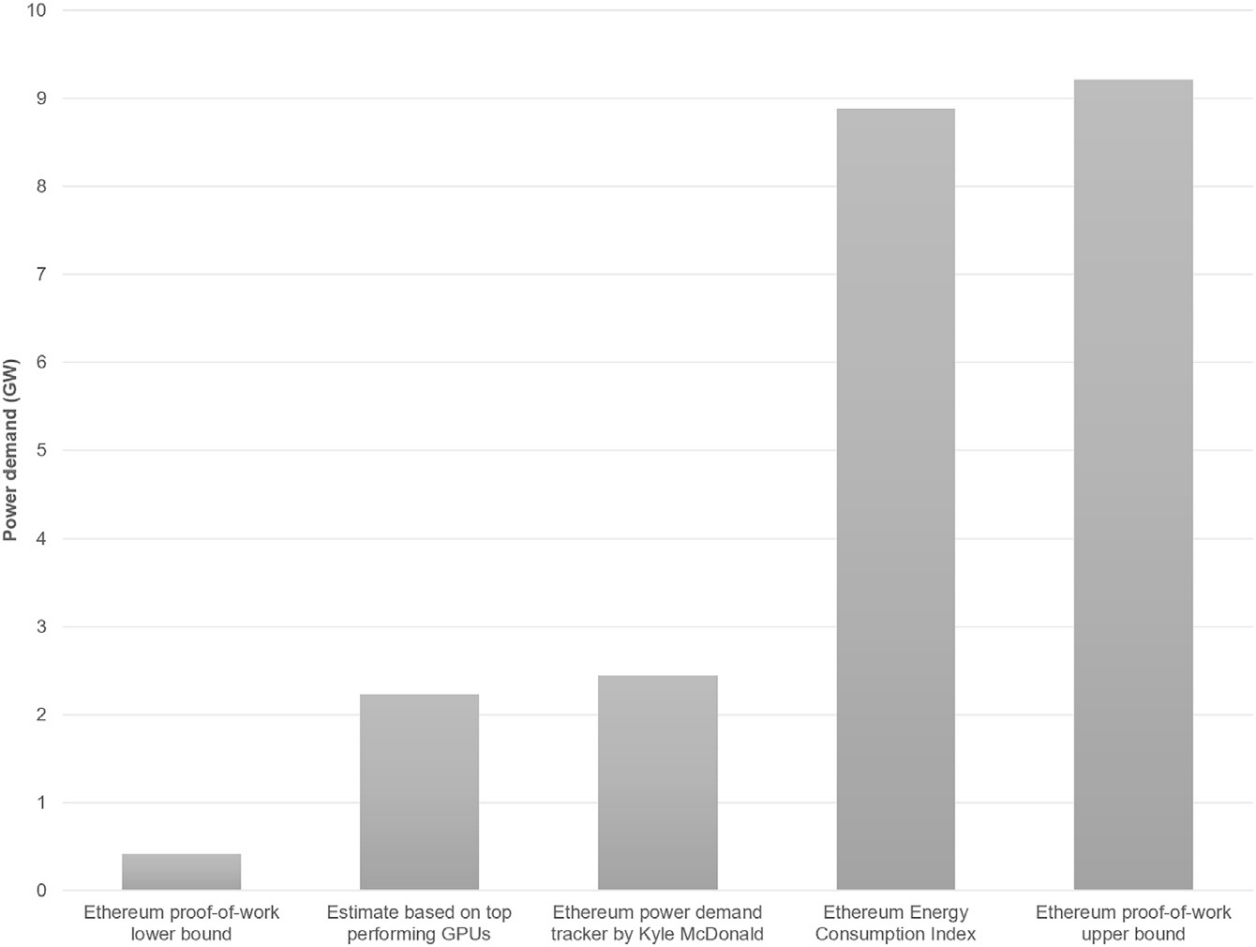
Various scenarios for Ethereum’s power demand prior to The Merge

A realistic estimate for the Ethereum network’s power demand before The Merge may be even higher, as a PoW mining network typically comprises a mix of device types, and additional energy costs may be incurred for cooling large batches of these mining devices. A tracker by Kyle McDonald estimated Ethereum’s power demand at 2.44 GW before The Merge.^11^ Moreover, the Ethereum Energy Consumption Index put this figure at 8.88 GW.^12^ The latter is approximately the maximum power demand Ethereum miners may have been able to afford, as they earned roughly 13,000 coins per day from mining. With Ether trading at around $1,700 USD in the week before The Merge, this amount translates to an available income of $22.1 million USD. With an electricity rate of 10 cents per kWh (commonly used in mining profitability calculators), the power demand of miners should not exceed 9.21 GW to avoid operating at a loss (also assuming no further expenses other than electricity). Figure 1 summarizes the various power requirement scenarios for PoW Ethereum.

Determining the remaining power demand of the Ethereum network after The Merge has its own challenges. In a PoS network, the power demand can be estimated by determining the number of connected network nodes and the electricity consumption profile of each node. However, because of the decentralized nature of the Ethereum network, there is no central overview of all connected nodes. Moreover, although one no longer has to account for the possibility of multiple mining facilities hiding behind an observed node in the network, estimated node counts still do not reveal the specifications of the underlying hardware. In Ethereum, the hardware requirements also depend on which combination of consensus and execution client is being used to operate a node. It has been suggested that running an Ethereum node should be possible with a Raspberry Pi 4GB running at just 8 W, though doing so is not recommended.^13^ At around 4,500 total nodes in the Ethereum network in the days after The Merge,^14^ assuming every node is a Raspberry Pi 4GB, this node count would translate to a power demand of just 36 kW. Using an enterprise server could increase the power requirement per individual device to 100–150 W,^13^ but it would still limit the total power demand of the Ethereum network to 675 kW, even if each node runs at 150 W.

Thus, in light of the previously calculated power requirement ranges, it appears likely that the Ethereum network reduced its power demand by at least 99.84% (considering the best-to-worst scenario) by switching from PoW to PoS (Table 1). At best, the total power demand reduction could reach even 99.9996% (in the worst-to-best scenario). Either way, The Merge most likely realized a significant decrease in the total power demand of the Ethereum network, as Ethereum on PoW required 619 to 255,833 times the electrical power Ethereum on PoS does. In absolute terms, the reduction in power demand could be equivalent to the electrical power requirement of a country such as Ireland or even Austria.

**Table 1:** Comparison of lower and upper bound power demand estimates for Ethereum before and after The Merge

Power demand scenario	Power demand (kW)	Reduction versus proof-of-work lower bound, %	Reduction versus proof-of-work upper bound, %
Ethereum PoS lower bound	36	−99.9914	−99.9996
Ethereum PoS upper bound	675	−99.8385	−99.9927

## Limiting factors

The reduction in power demand of the Ethereum network is, however, unlikely to reverberate globally. The devices previously used to mine Ethereum can still be repurposed. ASIC devices have limited options but could be used to mine the cryptoassets Ethereum Classic and EthereumPoW (an Ethereum spinoff that maintains the PoW mechanism). In the days after The Merge, both of these cryptoassets combined initially absorbed a quarter of Ethereum’s hashrate. One month after The Merge, the combined hashrate of Ethereum Classic and EthereumPoW continued to represent a fifth of Ethereum’s hashrate before The Merge. The GPUs used to mine Ethereum could be used on an even broader range of cryptoassets. This migration is likely to negate some of the potential reduction in global electricity consumption, although it must be noted that this effect is likely limited due to a significant reduction in the available miner income. For example, miners on Ethereum Classic can earn only approximately $0.5 million USD per day compared with the $22.1 million USD per day they could previously earn from mining Ethereum. This reduction in available income significantly limits the electricity expenses miners can afford.

The fact that GPUs can be repurposed outside cryptoasset mining further reduces the potential reduction of global electricity consumption. GPUs could be repurposed for other energy-intensive operations involving cloud computing, artificial intelligence, or simply for gaming a few hours per day. The latter would still save energy compared with using the same device 24/7 in the context of cryptocurrency mining, but because it is not possible to track former mining devices to their new purpose, the global reduction in electricity consumption as a result of The Merge is highly uncertain. Moreover, it has been suggested that Bitcoin miners have been able to take advantage of the data center space that became available after the reduction in Ethereum mining activities.^15^ Between 250,000 and 500,000 new Bitcoin mining devices reportedly remained unused as only a limited amount of rack space was immediately available.^16^

**Figure 2:**
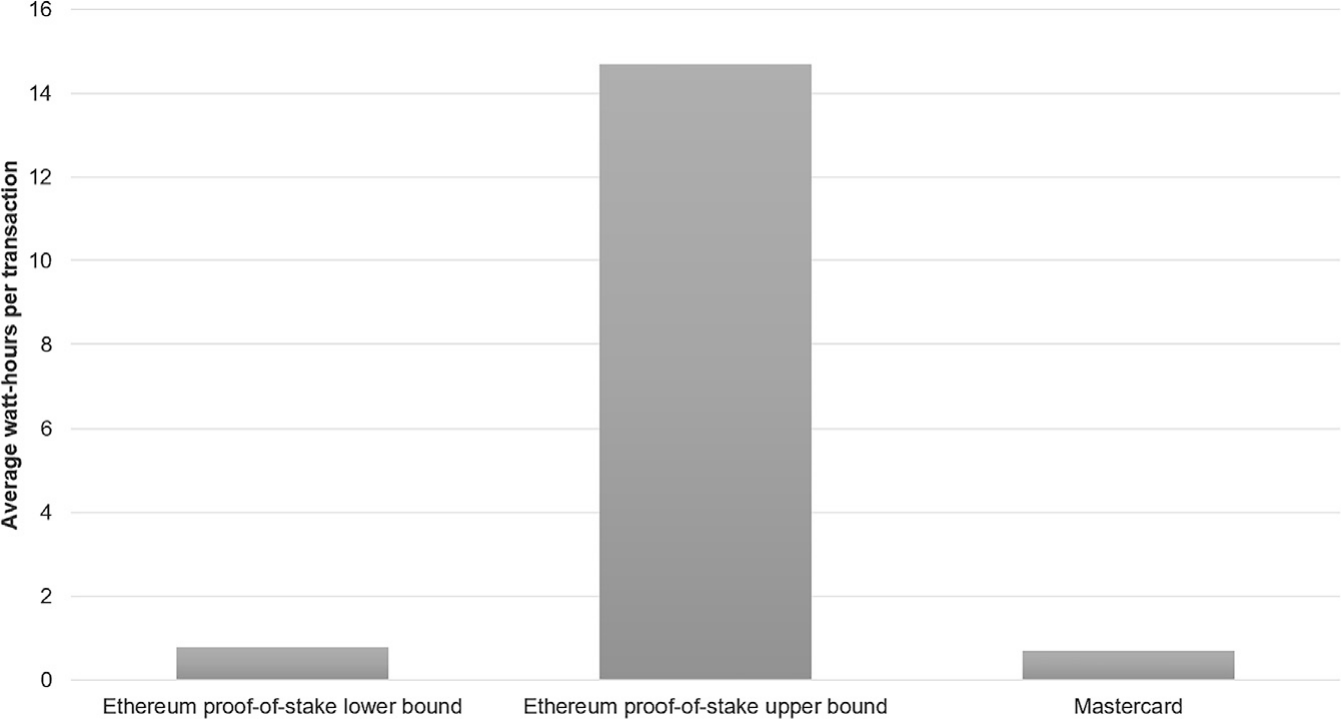
Energy efficiency of proof-of-stake (PoS) Ethereum and Mastercard transactions

Lastly, even though Ethereum likely did significantly reduce its own network’s power demand, it would still be premature for the Ethereum community to declare a complete victory over the sustainability concerns facing cryptoassets. The underlying blockchain technology functions by replicating data and processes over thousands of participating devices, thus increasing data redundancy and the associated (energy) costs of maintaining multiple copies. Consequently, the Ethereum network could remain relatively more energy inefficient than a more centralized alternative (Figure 2). For example, with Ethereum handling roughly 1.1 million transactions per day after The Merge, the average electricity consumed per transaction ranges from 0.8 to 14.7 Wh. In comparison, a Mastercard transaction consumes only 0.7 Wh on average.^17^ Decentralization continues to have a price, but proponents of cryptoassets and blockchain technology may be able to build a better argument that this feature is worth the additional energy costs.

## Replicating Ethereum’s success in other mineable cryptoassets

Even though Ethereum may have been exceedingly successful in reducing its power demand, Bitcoin, the largest polluter in the world of cryptoassets, continues to run on the energy-intensive PoW. The same is true for several smaller mineable cryptoassets such as Dogecoin and Litecoin. Moreover, the community behind Bitcoin has not worked on preparing a change to PoS, nor is there any substantial willingness in the community to do so. A campaign called Change the Code, launched by Greenpeace in March 2022, aimed at getting the Bitcoin community to replace its PoW mechanism. However, the campaign was met with hostility from the community^18^ as the underlying software’s lack of change (immutability) is seen as a key feature. The Bitcoin community also has a history of resisting substantial changes to the Bitcoin software, with one notable example being a past attempt to upgrade Bitcoin’s transaction-processing capacity. During the years 2015–2017, various community stakeholders pushed to increase the maximum amount of data allowed inside a block on the Bitcoin blockchain.^19^ This increase would have enabled Bitcoin to handle more than the handful of transactions that can be processed per second under the existing limit.^20^ In the end, only a small part of the community adopted the software version that would have changed the block size limit, which became a Bitcoin spinoff known as Bitcoin Cash. Any future attempt to replace PoW with PoS in Bitcoin might meet a similar fate, as the underlying network is decentralized and, therefore, does not have a central authority to enforce such a change.

Ethereum has, however, proven that it is not impossible to make the necessary changes to a live blockchain to make the software more sustainable. Moreover, it managed to do so despite resistance from various community stakeholders^21^ and concerns that PoS may lead to centralization in the Ethereum network.^22^ The success of the Ethereum community in overcoming these hurdles to have made The Merge happen suggests that, with the right capabilities and support, a similar success in changing the software may be achieved in Bitcoin. The widespread use of ASIC-based devices in Bitcoin mining^23^ also makes it more likely that any reduction in Bitcoin’s power demand would also be reflected at a global level as it would not be possible to repurpose these mining devices in the same way as the GPUs previously used to mine Ethereum. Future research should, therefore, focus on determining the key factors that contributed to the success of The Merge as uncovering these factors could enable a change from PoW to PoS in Bitcoin and other cryptoassets still using PoW.
